# Salt Water Drops Slide Faster: Ionic Modulation of Drop Friction

**DOI:** 10.1002/advs.202521659

**Published:** 2026-01-21

**Authors:** Dongho Shin, Rutvik Lathia, Chirag Hinduja, Hyunbae Cheon, Seongmin Park, Hans‐Jürgen Butt, Junwoo Park

**Affiliations:** ^1^ Department of Chemistry Sogang University Seoul Republic of Korea; ^2^ Max Planck Institute For Polymer Research (MPI‐P) Mainz Germany; ^3^ Center For Nano Materials Sogang University Seoul Republic of Korea

**Keywords:** drop friction, ions, solid‐liquid interface, surface

## Abstract

The friction of drops on solid surfaces governs processes in microfluidics, energy devices, and surface engineering. In recent years, it has been established that slide electrification on insulating substrates leads to a substantial increase in drop friction. Here we present experimental evidence suggesting that such spontaneous charging effects impede the motion of drops not only on insulating substrates, but also on conductive substrates coated with nanometer‐thin hydrophobic films. We demonstrate addition of salts reduces drop friction. On PFOTS‐coated Si wafers and thiol‐functionalized Au, increasing NaCl concentration from deionized water to ≥ 0.1 m enhanced droplet acceleration by 75%–85%, corresponding to a 13%–25% reduction in friction force. This observation goes beyond electrostatic drop retardation by slide electrification, which had only been identified for insulating films much thicker than 0.1 µm. This phenomenon occurs independently of ion species, substrate doping type, and grounding conditions, and is not explained by changes in interfacial energy, viscosity, or electrostatic interactions alone. Instead, rapid ionic redistribution and electrohydrodynamic interactions at the interface dynamically couple droplet ions with electronic charges in the conductor, reducing contact angle hysteresis. Our findings provide a new framework for tailoring hydrodynamic behavior through charge carrier engineering at nanoscale interfaces.

## Graphical abstract

Salt ions markedly accelerate water drops on conductive substrates coated with nanometer‐thin hydrophobic layers. Across diverse electrolytes and surfaces, higher ion concentration consistently lowers kinetic friction by reducing contact angle hysteresis. Rapid ionic redistribution dynamically couples to electronic charges through the thin dielectric, homogenizing interfacial potentials and suppressing dissipation during sliding.

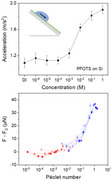

## 1 Introduction

Wetting phenomena pervade daily life and underlie a broad range of technologies, including printing, painting, coating, condensation, and evaporation in heat exchangers, the dispensing of herbicides and insecticides, and the functionality of waterproof fabrics, filters, soil, and flotation systems. The lateral adhesion or friction that resists a moving droplet is commonly quantified by the contact angle hysteresis (CAH)—the difference between the advancing and receding contact angles (Θ_a_ and Θ_r_)—which characterizes the pinning force at the three‐phase contact line. For instance, for a drop starting to move or sliding over a surface at low velocity, the friction is given by [1, 2, 3, 4, 5]:

(1) $$F_{0}=w\gamma_{L}k\left(\cos\Theta_{r}-\cos\Theta_{a}\right)\;$$

here, *w* is the width of the contact area of the drop and γ_*L*_ is the surface tension of the liquid. The factor *k*  ≈  1 depends on the drop shape and distribution of the CA along the contact line [5, 6, 7, 8]. Despite its central importance, the microscopic physical origin of CAH and its dominant dissipation pathways remain incompletely understood.

In dynamic wetting, a central question is what governs the CAH and the associated energy dissipation during drop motion. Multiple factors are known to contribute: surface roughness, chemical heterogeneity, elastic compliance of the solid, and chemical or structural adaptation of the surface can all play a role [9]. Even on smooth, homogeneous, rigid, and chemically inert surfaces, however, water often exhibits substantial CAH, indicating that an additional mechanism must be at work. Indeed, a major mechanism identified in recent years is spontaneous electrostatic charging at moving contact lines—commonly known as sliding or contact electrification. When water droplets slide over insulating hydrophobic surfaces, they deposit electric charge onto the solid and become oppositely charged themselves [10, 11, 12, 13, 14, 15, 16, 17, 18]. The resulting Coulomb interactions between charges in the drop and on the substrate impede the droplet's motion, thereby increasing frictional dissipation [19, 20, 21]. Three effects can be discriminated: long‐range coulomb forces [19], electrowetting [22], and an increased surface energy of the solid caused by the surface charges [20]. These findings established electrostatics as a central, yet previously overlooked, contribution to drop friction.

It has been assumed that the additional resistance on the drop due to slide electrification is limited to thick insulating surfaces. This assumption was based on two arguments: First, the electric field of deposited charges is shielded on a length scale equal to the thickness of such a layer, *d*. Second, the electrical resistance of nm thick layers is not sufficient to maintain a significant voltage on relevant time scales. As a result, conductive substrates coated with molecularly thin hydrophobic layers have been widely regarded as electrostatically inert in dynamic wetting. Here, we show a counter‐intuitive discovery: adding salt to the droplet—which typically increases viscous dissipation—actually accelerates its motion. This finding represents a significant shift in our understanding of how ionic species modulate interfacial friction at the nanoscale. We demonstrate that this phenomenon cannot be explained by changes in interfacial energy or bulk viscosity alone. Instead, we propose a new physical framework: the dynamic coupling between ions in the liquid and electronic charges in the substrate across a nanometer‐thin dielectric. Our results provide a foundation for precisely controlling liquid dynamics through ionic‐electronic coupling at nanoscale interfaces.

## 2 Results and Discussion

### 2.1 Salt‐Induced Changes in Kinetic Drop Friction

**FIGURE 1:**
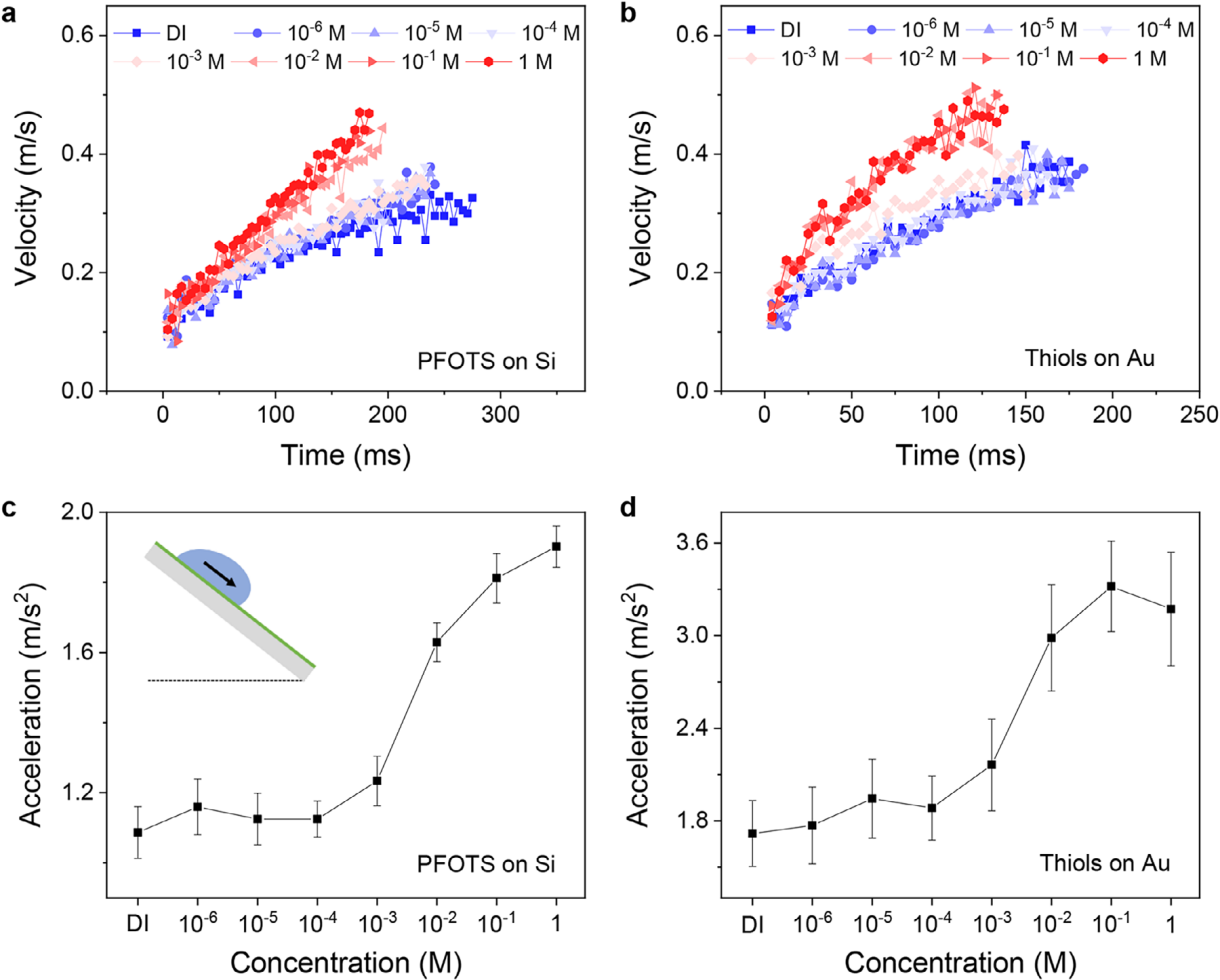
Effect of NaCl concentration on kinetic friction on SAMs‐coated conductive surfaces. (a,b) Velocity and (c,d) acceleration of water/NaCl droplets were analyzed as droplets slide down (a,c) PFOTS‐coated Si surfaces (n‐type, *p* ≤ 0.005 Ω·cm) and (b,d) hexanethiol‐coated Au surfaces. All experiments used 50° tilt angles and 37 µL water/NaCl droplets.

In contrast to previous experiments on slide electrification, we studied electrically conducting substrates. These substrates were only covered by a molecular hydrophobic layer. As examples, we tested drop sliding on highly conducting Si wafers (n‐type, *p*≤ 0.005 Ω·cm) coated with perfluorooctyltrichlorosilane (PFOTS) and gold coated with monolayers of n‐hexanethiol (CH_3_(CH_2_)_n‐1_SH). Water drops sliding down these surfaces slide faster when adding salt (Figure 1a,b). The addition of NaCl accelerated the water droplets. Increasing the salt concentration increased acceleration (Figure 1c,d) and decreased kinetic drop friction. t = 0 is the moment the water drops fall on the surface and start to slide. The acceleration was obtained by fitting the first 100 ms of the velocity‐vs.‐time curves linearly. This short timeframe ensures that the measurement is completed before significant evaporation or salt crystallization can occur at the contact line [23]. Comparing deionized (DI) water with 1 m NaCl droplets, the acceleration increased by 75%. The main increase in acceleration was observed around 3 mM salt.

The resulting acceleration was then used to estimate the kinetic friction force (*f*) using Newton's second law. For the droplet slides under gravity on an inclined plane, the net force is given by [19, 24, 25, 26] *F*  =  *ma*  =  *mgsin*θ − *f*, where θ is the tilt angle of the surface, *m* is the mass of the droplet and *a* is the measured acceleration from the linear fit. Here, we neglect the additional inertia of the drop due to a rolling component [19]. For 1 m NaCl drops compared to deionized water, an increase in acceleration of 75% corresponds to a 13% decrease in kinetic friction on the PFOTS‐coated Si surface. Hexanethiols (C_6_) on the gold surface showed an 85% increase in acceleration, corresponding to a 25% decrease in the friction.

### 2.2 Influence of Salt Species, Substrate Conductivity, History, Electrical Grounding, and the Thickness of Insulating Layers

**FIGURE 2:**
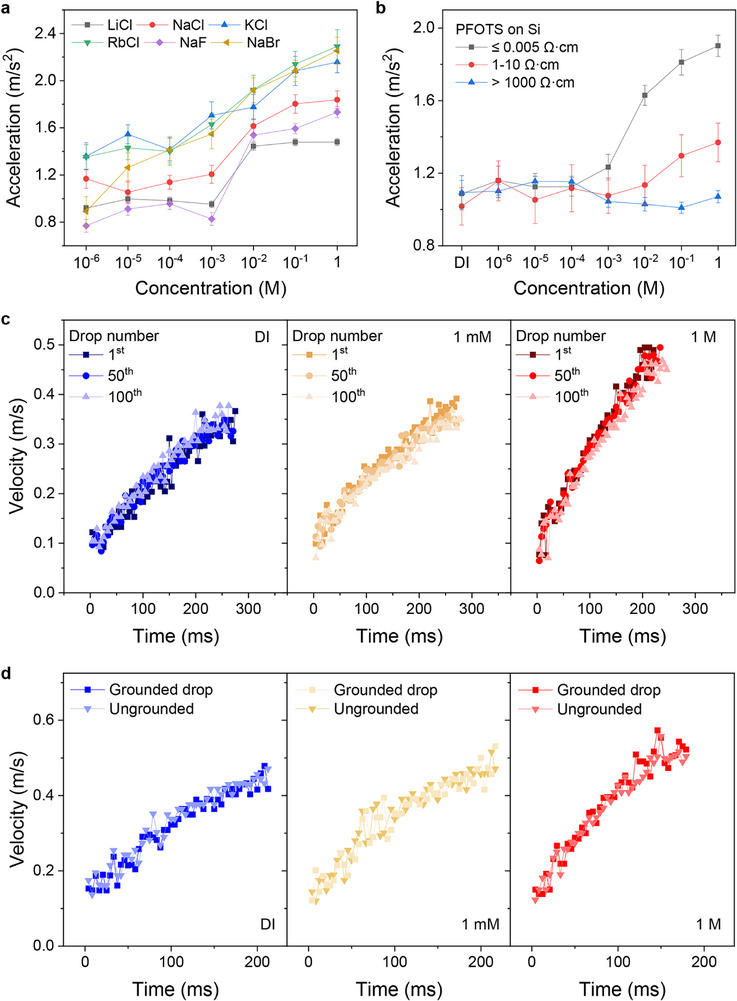
Influence of substrate conductivity, type of salt, drop number, and grounding on drop motion. (a) The acceleration phenomenon is not limited to specific electrolytes but appears in most electrolyte solutions. (b) Accelerating droplets on PFOTS‐coated Si surfaces with different substrate resistances confirmed that this phenomenon occurs only when the substrate exhibits high conductivity. (c) Measuring droplet sliding velocity, regardless of the number of droplets, showed no variation, confirming the absence of hysteresis. (d) Droplet sliding velocity remained unchanged regardless of grounding with a wire, confirming that electrostatic interactions do not drive this mechanism. All experiments used 50° tilt angles and 37 µL droplet volumes, and n‐type Si wafers were used.

The ion‐driven change in friction is observed across various monovalent ions tested, suggesting a general trend. Minor quantitative differences between ion species may be attributed to differences in their diffusion coefficients, which affect the dynamic screening rate. We additionally tried LiCl, KCl, RbCl, NaF, and NaBr (Figure 2a; Figure S3). Independent of the specific type of salt, kinetic friction decreases with increasing ion concentration. This phenomenon was observed regardless of the carrier type of the semiconductor; n‐type and p‐type Si wafers (*p* ≤ 0.005 Ω·cm) coated with PFOTS SAMs (Figure S4). Both carrier type showed a similar increase in acceleration with an increase in the salt concentration.

To determine whether the conductivity of the substrate affects the salt dependence of drop sliding, we tested Si wafers with different doping concentrations while maintaining the PFOTS coating (Figure 2b; Figure S5). Compared to the change in friction observed on a highly conductive Si wafer (*p*≤ 0.005 Ω·cm), the degree of change was smaller on a silicon substrate with a resistivity of 1–10 Ω·cm. No influence of salt was observed on a silicon substrate with a resistivity of ∼1000 Ω·cm. Since the friction change was measured on the same substrate by varying only the liquid, this trend confirms the electronic effect regardless of any static surface roughness variations caused by doping. The fact that the drop sliding on PFOTS‐coated Si wafers is only affected by salt for highly conducting substrates excludes several hypotheses to explain the effect. Since all samples have the same surface chemistry, neither defects existing on the solid surface, adaptation or deformation of the solid substrate, nor the influence of nanobubbles at the solid‐liquid interface [27] can account for the salt‐induced acceleration of the drop.

The drop in acceleration by salt is not caused by a change in viscosity because the viscosity increases with salt concentration. The addition of salt should lead to more, not less, viscous energy dissipation. At 20°C, the viscosity of distilled water is 1.00 mPa·s, while that of a 1 m NaCl solution is 1.02 mPa·s. This value corresponds to an increase of approximately 2%. Furthermore, the acceleration by salt was not observed on non‐conductive hydrophobic surfaces (e.g., lotus leaf, resistive‐Si wafer, PVDF, Teflon, polystyrene surface in Figure S7). In these surfaces, the accelerations were similar for NaCl concentrations varying from 10^−6^ to 1 m.

During drop sliding on thick hydrophobic layers, electric charges remain on the solid surface. For a series of drops, these surface charges change the motion of the following drops. As a result, drop motion depends on the number of drops in a series [19] and the interval time. Only if the discharging time is shorter than the interval between drops, all drops move similarly. To determine whether the surfaces remain charged after a given interval (one second), we monitored the drop velocity by consecutively dispensing 100 water droplets on a PFOTS‐coated silicon wafer (resistivity≤ 0.005 Ω·cm, Figure 2c). From DI water to 1 m NaCl solution, no significant difference in the drop motion was observed for the first, 50th, and 100th drops (Figure 2c; Figure S8). The velocity of a drop is not influenced by previous drops. The observation that all drops in a series slide at a similar velocity as subsequent drops (Figure 2c; Figure S8) indicates that surface charges are neutralized in well under 1 s.

This observation implies that the nanometer‐thick dielectric layers have a low resistivity. Estimating the resistivity using continuum theory and bulk parameters, the electrical relaxation time (RC time) of a dielectric layer is given by τ_*S*_ = ρ_*S*_ ε_*S*_ε_0_. Assuming the relative permittivity ε_*S*_ =  4 and using τ_*S*_ ≪ 1 s, the effective resistivity ρ_*S*_ of the dielectric layer must be lower than 2.8 × 10^10^ Ω⋅m. To validate this, we measured the macroscopic conductivity of the C_12_ thiol monolayer and found κ ≈ 10^−13^ S/cm. (see Experimental Section). This experimental value corresponds to a resistivity consistent with the requirement for rapid discharge, confirming that the nanometer‐thin film allows sufficient charge transport. However, polymers such as polyethylene and polystyrene have a much higher resistivity. This estimation ignores the possibility that a 1 nm thick film may have different properties from a bulk polymer. Furthermore, any defect or heterogeneity in the film would drastically increase its conductivity. The relatively low resistivity of these thin layers indicates a higher degree of heterogeneity compared to thicker films. This fact is to be expected because any defect in the top molecular layer exposes the gold or silicon surface. In the case of thick alkane or polymer layers, a disorder exposes the alkane or polymer.

**FIGURE 3:**
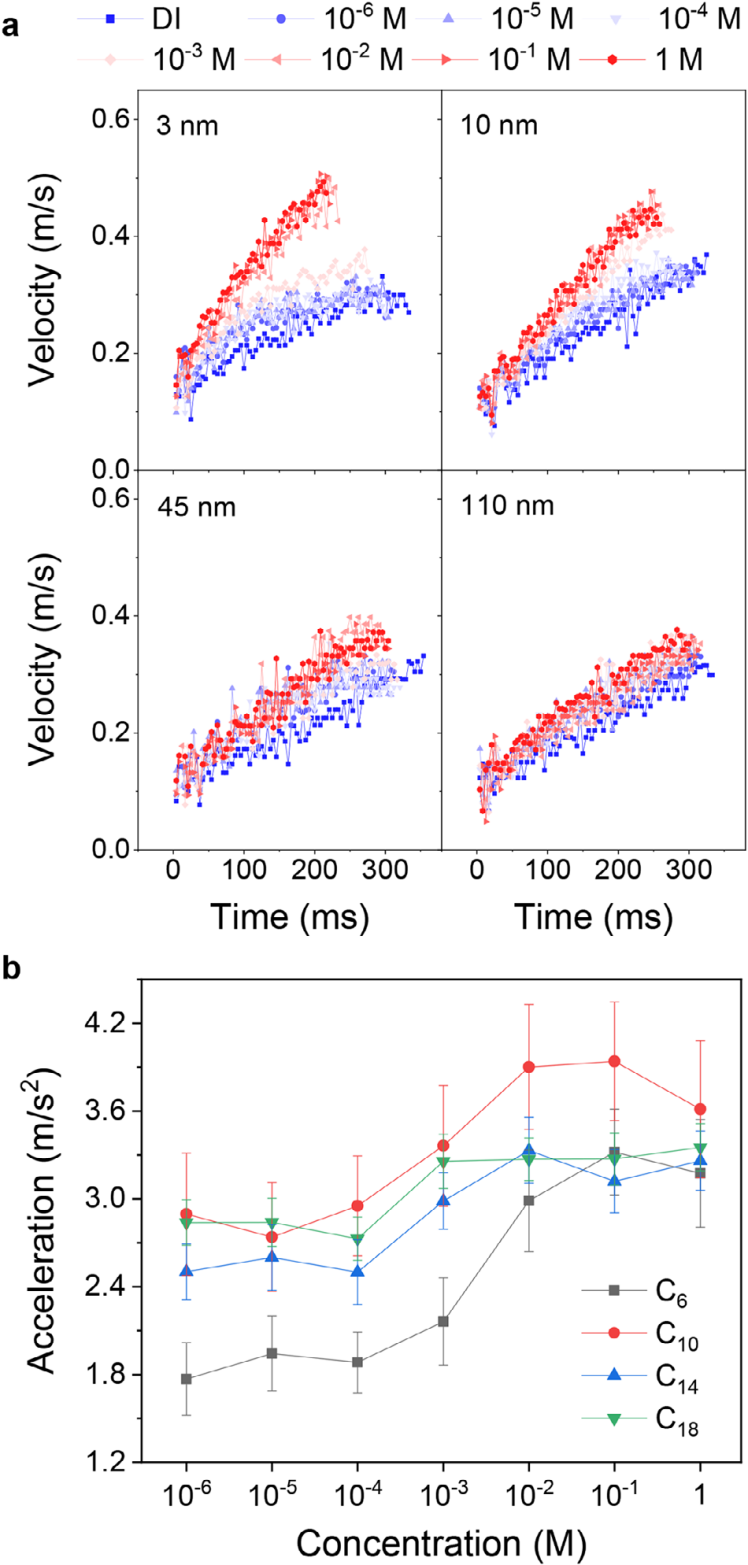
Effect of insulating layer thickness on droplet velocity. Velocity measurements on functionalized surfaces. (a) Velocity measurements were performed on PFOTS‐coated Si surfaces (n‐type, *p* ≤ 0.005 Ω·cm) with oxide layers of 3, 10, 45, and 110 nm. (b) Velocity measurements were conducted after depositing thiols with varying carbon chain lengths on a gold surface. A comparison of results from both surface types confirmed that the insulating layer thickness significantly influences velocity variations across different concentrations. All experiments used a 50° tilt angle and a 37 µL droplet volume.

For the thiols on gold, electron tunneling can also increase the conductivity [28, 29]. As expected, we observed that the salt‐induced acceleration disappeared as the insulator thickness increased. As the silicon oxide layer on the conductive silicon substrate increased in thickness (Figure 3a) and the length of thiol molecules on the gold surface increased (Figure 3b; Figures S11 and S12), the change in kinetic friction decreased. An increase in thickness lowers the conductivity of the organic insulating layer and hinders the neutralization of ions.

On thick insulating layers, the long‐range Coulomb force between the drop and the deposited charges could slow down drop motion [19]. To demonstrate that long‐range Coulomb forces are unlikely to explain the observed acceleration with salt, we grounded the drop with a Cu wire of 0.3 mm diameter spanned parallel to the surface at a height of 2 mm. We compared the friction with and without grounding (Figure S9). Electrical grounding did not affect the drop motion (Figure 2d). We still observed the same increase in acceleration in the concentration range of DI water to 1 m NaCl (Figure S10). Therefore, long‐range Coulomb forces between the water drop and the substrate are not the cause of this phenomenon. This conclusion agrees with the electrostatic theory, which predicts that charges on the free solid surface are screened by the grounded metal with an effective screening length equal to the thickness of the insulating layer *d*. At distances much larger than *d*, the field decreases steeply because the charge, together with the image charge in the metal, appears as a dipole.

### 2.3 Change in Dynamic Contact Angles of a Sliding Drop

**FIGURE 4:**
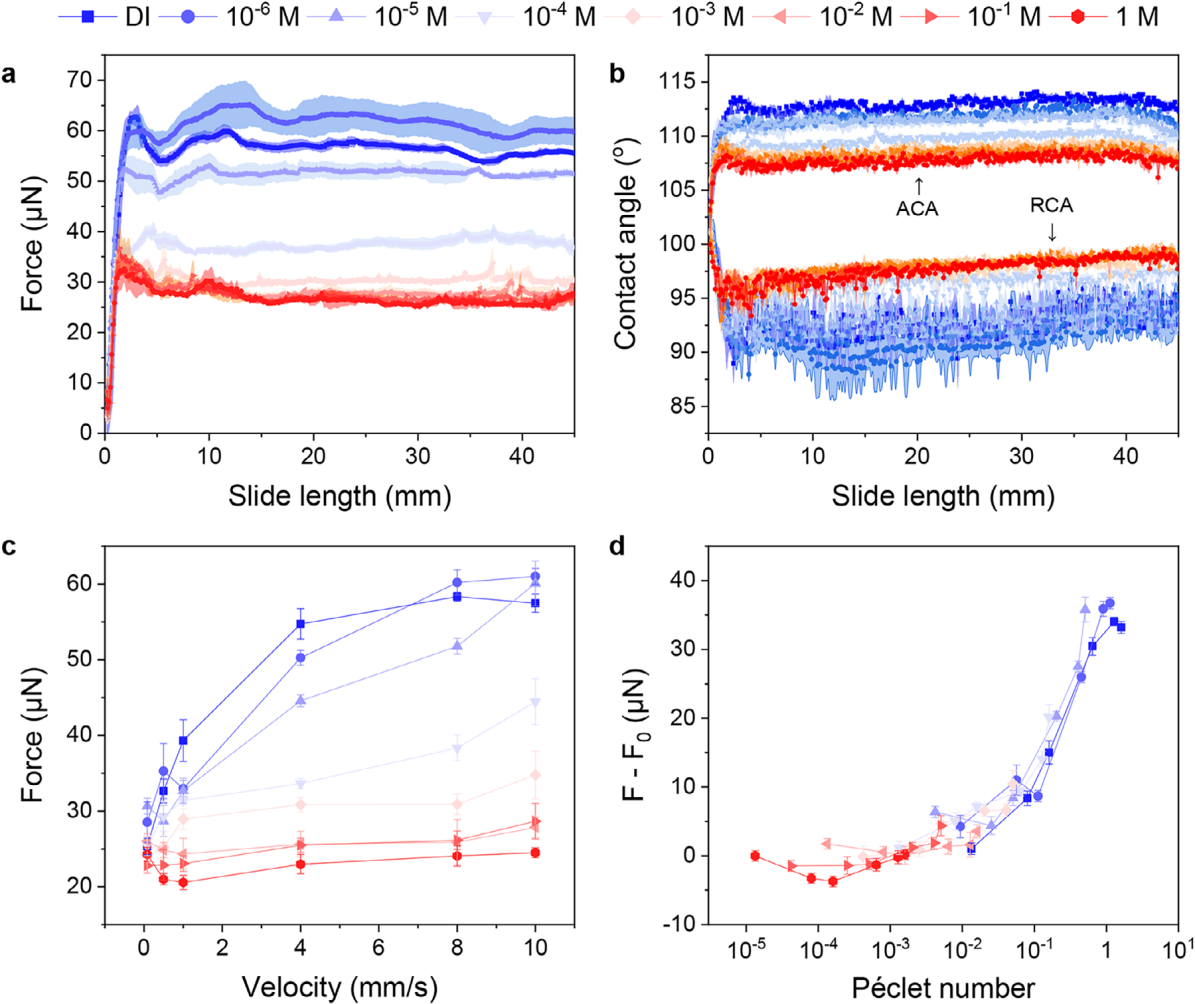
Measurement of kinetic friction and dynamic contact angles. Results from the drop friction force instrument (DoFFI) method for SAMs of dodecanethiol (C_12_H_25_SH) on gold. (a) Experimental results for droplet friction forces measured for different concentrations of 20 µL KNO_3_ droplets at 8 mm/s velocity. (b) Corresponding advancing (ACA) and receding contact angles (RCA). (c) Kinetic friction forces obtained from DoFFI experiments at different velocities for the concentration range from DI water to 1 M KNO_3_. Dynamic friction force values were obtained from the average value of force once the droplet starts sliding over the surface (> 5 mm sliding distance). (d) Additional friction forces, *F* − *F*_0_, change with Péclet number. *F* is a friction force, and *F*_0_ is a reference force (for 1 M KNO_3_ at 0.083 mm/s). For the calculation of Péclet number, concentration of DI water was taken as 10^−6^ M (due to contamination) and 2 ×10^−6^ M for 1 µM concentration (due to additional contribution from DI water).

Having ruled out long‐range Coulomb forces as an explanation for the higher mobility of drops at high salt concentration, we now focus on effects acting near the contact line. “Near” refers to a length scale of ≈10 µm, which is the resolution with which we determine the contact angles using a side‐camera. Drop friction and the contact angles are intimately related (Equation 1). To gain more insight, we measured friction, advancing, and receding contact angles simultaneously. These measurements were carried out using the drop friction force instrument (DoFFI) [30]. In DoFFI, a sessile drop is kept in place with a glass cantilever, which acts as a spring, while the substrate underneath the drop moves at constant velocity. The force is obtained from the deflection of the spring. Contact angles are determined from side‐view videos. Using DoFFI, we compared the friction force acting on drops sliding on the monolayers of dodecanethiol (C_12_H_25_SH) on gold (Figure 4a) and PFOTS‐coated conductive Si wafers (Figure S13). With an increase in salt concentration, the kinetic friction of water drops decreased (Figure 4a). The friction force traces exhibit natural fluctuations due to the microscopic stick‐slip motion of the contact line, which were captured by our high‐resolution tracking. Moreover, the advancing contact angle (ACA) decreased by an average of about 6° from 113° in DI water to 107° in 1 m KNO_3_ (Figure 4b). The receding contact angle (RCA) increased by 7° from 92° in DI water to 99° in 1 m KNO_3_. Contact angle hysteresis decreased with the addition of salt. Hence, the friction force decreased (Equation 1).

To interpret these changes in contact angle, we first eliminate two effects, which are important on thick insulating surfaces: Electrowetting and charge deposition. We start with Young's equation. Assuming that the forces acting horizontally on the contact line balance in steady state motion, Young's equation would describe the local contact angle Θ according to γ_*L*_ cos Θ = γ_*S*_  − γ_*SL*_. Here, γ*_L_* is the surface tension of the liquid, γ*_S_* is the surface energy of the free solid surface, and γ*_SL_* is the solid–liquid interfacial energy near the contact line. As mentioned above, one possible effect leading to drop retardation is a decrease in the solid‐liquid interfacial energy caused by an electric potential across the insulating layer [20]. This effect is called electrowetting [31]. However, electrowetting and the resulting decrease in γ*_SL_* should decrease both contact angles, advancing and receding; the receding contact angles were observed to increase. Thus, electrowetting does not seem to play a significant role.

The second possible contribution to the low receding contact angle at low salt concentrations is charge deposition. If charges are deposited at the solid‐air interface, the motion of the drop would slow down due to an increase in solid surface energy [20]. This increase in solid surface energy is proportional to the charge density squared (∝σ^2^). Here, σ is the charge density right behind the drop. The addition of salt eliminates the charge transfer. Such a reduced charge transfer has been reported previously. When drop charging is measured as a function of salt concentration, a decrease is typically observed for salt concentrations above 1 mM [12, 16, 32, 33]. The addition of charged molecules such as fluorophores [34] or surfactants also reduces the drop charge [35]. This hypothesis can explain why drops slide faster or experience a lower friction force at high salt concentrations. However, this effect cannot explain the decrease in advancing contact angle when adding salt.

These results can be interpreted by assuming that the addition of salt eliminates the electrostatic contribution to contact angle hysteresis, thereby reducing drop friction. We propose that a high salt concentration leads to a more homogeneous solid‐liquid interface, which reduces contact angle hysteresis. Structural inhomogeneities in the molecular packing of the organic layer lead to variations in the local charges and dipoles. One cause of inhomogeneity is the surface charge itself. The typical zeta potential of hydrophobic surfaces in an aqueous electrolyte solution at salt concentrations ranging from 0.1 to 10 mM is between −30 and −60 mV [36, 37]. Using the Grahame equation, we can estimate the charge density at the solid‐liquid interface σ_*SL*_. For low surface potentials ψ_0_ it is given by, σ_*SL*_ = ε_*L*_ε_0_ψ_0_/λ . The Debye length λ characterizes the length scale over which electric fields in electrolytes are screened. For a monovalent salt of concentration c_0_, it is given by $\lambda =\sqrt{\varepsilon_{L}\varepsilon_{0}k_{B}T/2c_{0}e^{2}}$. Here, ε_*L*_ is the relative dielectric permittivity of the liquid, ε_0_ =  8.85  ×  10^−12^ F/m is the vacuum permittivity, *k_B_* is the Boltzmann constant, *T* is the absolute temperature, and *e* is the unit charge. For aqueous electrolyte at room temperature $\lambda \approx 0.3\;\mathrm{nm}/\sqrt{c_{0}}$, in which *c*_0_ should be inserted in mol/L. Typical charge densities of −1 to −10 mC/m^2^ are obtained. On average, the surface charges are spaced 10–20 nm apart at 0.1 mM salt and 2–4 nm apart at 10 mM. This estimation supports the idea that surfaces are homogeneous on a length scale larger than 1 nm at high salt concentrations, whereas at low salt concentrations, they only appear homogeneous on a length scale much larger than 10 nm. Furthermore, inhomogeneities of the charge distribution at the interfaces are smoothed out on a length scale of the order of the Debye length. For example, at 10 mM salt concentration, any variation of electric potential on a scale above 3 nm should be equilibrated by ions from the solution. At 0.1 mM salt concentration, the corresponding equilibration only happens on a length scale of 30 nm, leaving a more inhomogeneous interface.

**FIGURE 5:**
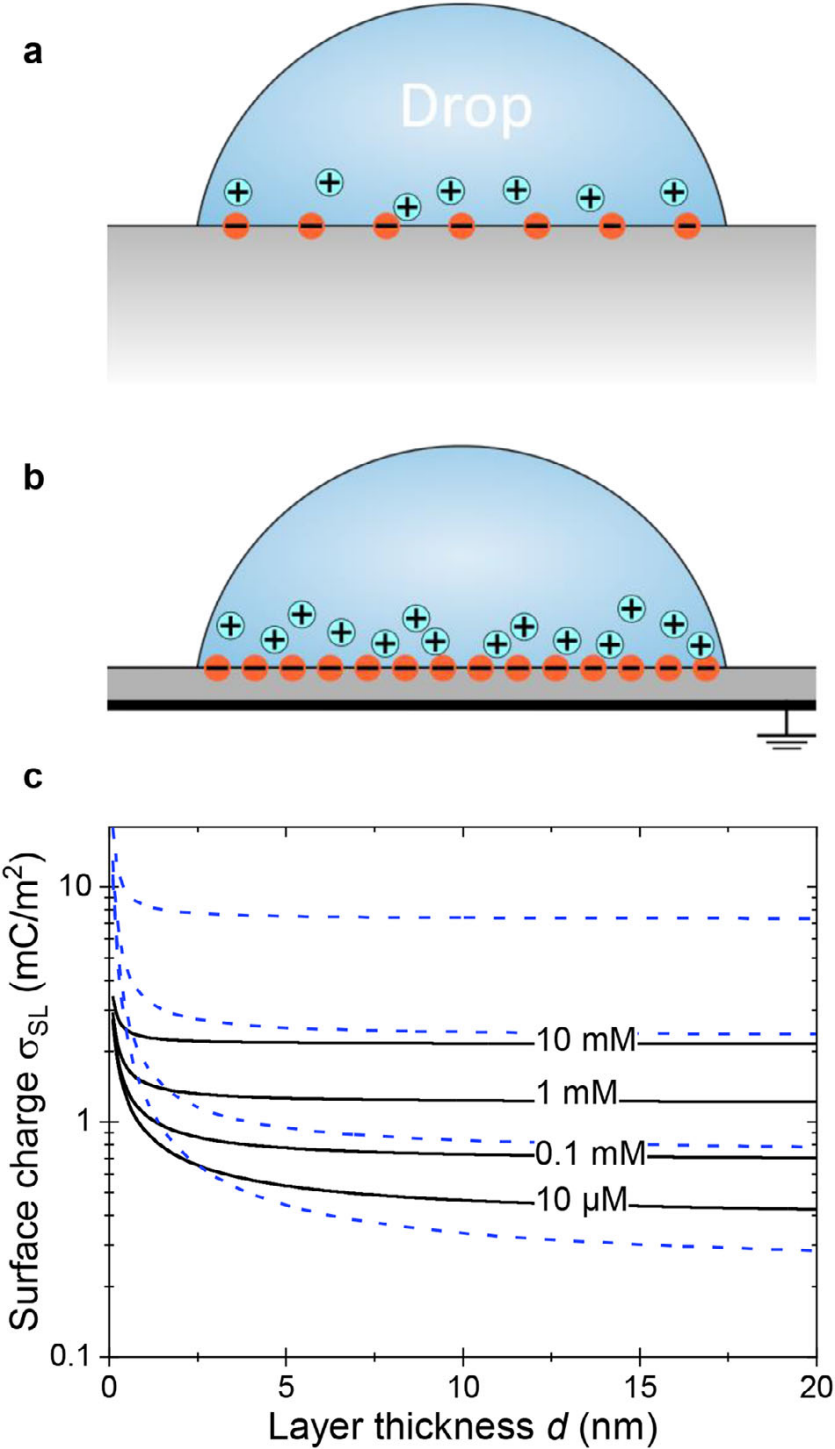
Drop configuration on dielectric layers. Schematic of a sessile drop on (a) a thick and (b) a thin dielectric layer. The ions forming the electric double layer are shown. In this case, the surface charge σ_*SL*_ is negative, and the diffuse layer consists of cations. Due to the screening by the grounded metal, the spontaneously formed surface charge density is higher for thin dielectric films. (c) Surface charge density σ_*SL*_ vs. the thickness of the dielectric layer calculated for ε_*S*_ = 4 and ε_*L*_ = 78 for different concentrations of a monovalent background salt calculated with Equation (4) (black lines). Equation (4) is limited to low surface potentials. The lines were calculated assuming that σ_*SL*_ ψ_0_ =  2  ×  10^−5^ N/m remains constant. The blue dotted lines are for the constant surface potential of ψ_0_ =  0.03 V calculated with Equation (2). The blue dotted lines were also calculated for salt concentrations of 10 µM to 10 mM from bottom to top.

Why is interface homogenization by salt only important for thin insulating layers? One possibility is that, for conducting substrates, such inhomogeneities may be more pronounced because the underlying metal screens electrostatic effects. To support the hypothesis that electrostatic potential variations may be amplified on conducting substrates due to image charges, we demonstrate that, for very thin insulating layers, the proximity of the metal increases the total charge density at the solid‐liquid interface σ_*SL*_ (Figure 5) [38]. We calculate the relationship between surface charge density and surface potential ψ_0_. For an infinitely thick dielectric, this relation is known as the Grahame equation [39]. To account for the grounded metal, the Grahame equation needs to be extended:

(2) $$\sigma_{SL}=\varepsilon_{S}\;\varepsilon_{0}\frac{\psi_{0}}{d}+\sqrt{8c_{0}\varepsilon_{L}\varepsilon_{0}k_{B}T}\sinh\frac{e\psi_{0}}{2k_{B}T}\;$$

The first term has to be added to account for the screening by image charges in the metal. The second term is equal to the expression in the Grahame equation. For low surface potential, this expression simplifies to:

(3) $$\sigma_{SL}=\varepsilon_{0}\;\psi_{0}\left(\frac{\varepsilon_{S}}{d}+\frac{\varepsilon_{L}}{\lambda }\right)\;$$

This modified Grahame equation shows that for a given surface potential, the surface charge density increases as the thickness of the dielectric layer decreases (Figure 5c, blue dotted lines). It is energetically more favourable to bind ions to the surface because their potential is screened by the underlying metal by image charges.

When changing the concentration of background salt, in general, surface charge and surface potential will change. Adding ions tends to decrease ψ_0_ and increase σ_*SL*_ [36, 40]. Constant surface potential is not a realistic boundary condition. More realistic is to assume that the product σ_*SL*_ψ_0_ remains constant when changing the salt concentration. σ_*SL*_ψ_0_ represents the chemical energy of the reaction, causing charging in the first place. It is given in units of J/m^2^ or N/m [41, 42, 43]. Assuming constant σ_*SL*_ψ_0_ and in the low potential limit, we get

(4) $$\sigma_{SL}=\sqrt{\varepsilon_{0}\sigma_{SL}\psi_{0}\left(\frac{\varepsilon_{S}}{d}+\frac{\varepsilon_{L}}{\lambda }\right)}\;$$

and

(5) $$\psi_{0}=\sqrt{\frac{\sigma_{SL}\psi_{0}}{\varepsilon_{0}\left(\frac{\varepsilon_{S}}{d}+\frac{\varepsilon_{L}}{\lambda }\right)}}\;$$

Thus, also for constant σ_*SL*_ψ_0_ the surface charge density will substantially increase when the thickness of the dielectric layer decreases below ≈2 nm (Figure 5c black lines).

Assuming a constant charging propensity, Equation (4) predicts that σ_*SL*_ increases by a factor of ∼3 when the dielectric thickness decreases from 10 to 1 nm. Since the electrostatic contribution to contact line pinning is expected to scale with σ^2^, this corresponds to a ∼12× increase in the potential energy landscape associated with spatial heterogeneity. This trend matches the measured friction force reduction of ∼25% and the CAH suppression of ∼15° with salt addition, implying that ionic screening of charge fluctuations dynamically smooths the energy landscape.

### 2.4 Velocity Dependence

To distinguish kinetic and equilibrium effects, we conducted DoFFI experiments at varying velocities. Figure 4c shows the average dynamic friction force of 20 µL drops once they were in continuous motion. For low velocities (≤100 µm/s), the friction force on the drop remained low (≈26 µN) and was barely affected by the salt concentration. Increasing the velocity caused the friction force to increase twofold at low salt concentrations (≤0.1 mM), whereas it remained constant for salt concentrations of ≥100 mM.

To better understand the origin of the additional friction force at high velocity and low salt concentration, it is helpful to plot it against the Péclet number, *P_e_* (Figure 4d). In these plots, we plotted the force measured for a 1 m KNO_3_ salt solution at a velocity of 0.08 mm/s (denoted by *F*_0_), which has been subtracted. *F*_0_ was subtracted to consider other effects, e.g., heterogeneity or roughness. The Péclet number measures the importance of advective over diffusive transport. Here, we define it as *P_e_* = *v*λ/*D* [44], where *D* is the mean diffusion coefficient of the ions (∼ 1.9 × 10^−9^ m^2^/s). At the front of the drop, a new interface is formed with a flow velocity that is equal to the velocity of the drop. An electric double layer forms spontaneously [45, 46]. The reverse occurs at the rear of the drop, where the electric double layer of the solid‐liquid interface is replaced by the solid‐air interface. During the formation of the electric double layer at the front of the drop, the ions must diffuse a distance characterized by the Debye length. When plotting the additional friction forces measured at various salt concentrations against *P_e_*, all curves fall onto one master curve. This indicates that relaxation processes related to rearrangements of the electric double layer near the contact line may cause the extra drop in friction.

The fact that the additional friction forces, *F* − *F*_0_, plotted vs. *P_e_* all fall on one master curve also allows an alternative interpretation. The salt dependence could also indicate that electric potential gradients in the lateral direction somehow cause a drop in friction. Since negative ions are continuously removed at the back side of the drop, a potential gradient may develop in the drop if the ions are not redistributed fast enough. The Péclet number can be seen as the characteristic time scale of diffusion relaxation divided by the time scale characterizing advection. Along the sliding direction, potentials relax with a characteristic time scale of τ_*d*_ = *L*λ/*D* [47, 48], where *L* is the length of the drop. Here, it was assumed that λ ≪ *L*. Advection would be characterized by τ_*a*_ = *L*/*v* . With *Pe*  = τ_*d*_/τ_*a*_ , the resulting Péclet number is identical to that obtained for electric double‐layer relaxation, though the interpretation differs. The Péclet number (*P_e_* = *v*λ/*D* ) serves as a metric for the competition between the droplet's sliding speed, representing convection, and the ionic relaxation speed, representing diffusion. A high *P_e_* suggests that ions cannot rearrange fast enough to screen the surface charges at the moving contact line, leading to a retarding electrostatic force. Conversely, adding salt reduces the Debye length (λ), effectively lowering *P_e_* and enabling rapid ionic equilibration, which minimizes this drag.

In summary, we have demonstrated that the addition of salts significantly reduces the kinetic friction of water droplets sliding on conductive substrates coated with nanometer‐thin insulating films. We conclude that this friction reduction is governed by the two mechanisms. First, electrostatic homogenization, as discussed in Section 2.3, allows high salt concentrations to screen the electric fields arising from surface defects on the thin dielectric. This creates a smoother electrostatic potential energy landscape for the contact line, reducing the baseline pinning force. Second, dynamic relaxation, discussed in Section 2.4, ensures the maintenance of this screened state depending on the dynamics. The Péclet number (*P_e_* = *v*λ/*D* ) characterizes this competition. At high salt concentrations (low *P_e_*), rapid ion redistribution prevents the formation of a retarding electrostatic drag. These findings highlight a novel form of ionic‐electronic coupling across nanoscale interfaces, offering a new framework for controlling wetting dynamics.

## 3 Experimental Section

### 3.1 Sample Preparations

#### 3.1.1 Materials

We used ultrapure water (18.2 MΩ cm; Milli‐Q Ultra‐Pure, Merck). All electrolyte solutions were initially prepared at 1 m concentration and subsequently diluted to the desired concentrations. LiCl (99.0%∼100.5%, Sigma–Aldrich), NaCl (≥99.0%, Samchun chemicals), KCl (≥99.0%, Sigma–Aldrich), RbCl (≥99.0%, Sigma–Aldrich), NaF (≥97.0%, DAEJUNG), NaBr (99.0%∼101.0%, DAEJUNG), and KNO_3_ (≥99.0%, DAEJUNG)

#### 3.1.2 Deposition of PFOTS SAMs

The PFOTS‐coated Si surfaces (*p* ≤ 0.005 Ω·cm) were prepared using chemical vapor deposition (CVD). Clean Si wafers were activated by 100% O_2_ plasma treatment for 10 min. The wafers were then placed in a vacuum desiccator containing a small glass bottle with 0.5 mL of 1H,1H,2H,2H‐perfluorooctyltrichlorosilane (Sigma–Aldrich). The desiccator was evacuated to <100 mbar and kept under vacuum for 10 min at room temperature. After 30 min, the samples were annealed at 100°C for 10 min to remove any untreated fluorosilane. Subsequently, they were rinsed with isopropanol (≥99.5%, DAEJUNG) to remove unbound silanes. The thickness of the silicon oxide layers was measured using a spectroscopic ellipsometer (Alpha SE, J. A. Woollam Co. Ltd.).

#### 3.1.3 Deposition of Thiol SAMs

Thiols‐gold surfaces were prepared by immersing fresh gold substrates in 1 mM alkanethiolates (97%, Sigma–Aldrich)/ethanol (≥99.5%, Sigma–Aldrich) solution for 24 h. Then the surfaces were taken out and rinsed with fresh ethanol (≥99.5%, Sigma–Aldrich) to remove unbound thiols. Conductivity of C_12_ thiol was found to be 10^−13^ S/cm. A 20 µL droplet of 1 mM NaCl solution was placed on the C_12_‐thiol monolayer surface, and a pulse voltage of 1 V was applied between the droplet and the underlying gold layer; the steady‐state current was I_∞_​ = 0.20 µA. Taking the droplet base diameter as 5 mm (Area, A = πr^2^ = 1.96 × 10^−5^ m^2^) and the SAM thickness d = 1.6 nm, the conductivity was calculated from κ = I_∞_​d/(VA), yielding κ ≈ 10^−13^ S/cm.

### 3.2 Measurement of Sliding Drop

A home‐built tilted plate setup measured the droplet velocity. A syringe needle, connected to a Fusion 720 peristaltic pump (Chemyx, Inc.), automatically placed the drops on the tilted surface. The 5 mm height between the syringe needle and the surface ensured proper droplet release. A camera recorded the droplets (240 frames) from the side as they slid after contacting the surface. A custom‐developed code analyzed the side‐view videos of the sliding droplets to measure the velocity of the front contact line. The linear fit for determining the initial acceleration was performed from t = 0 to 100 ms. All measurements took place at 25°C ± 1°C and 40% ± 10% relative humidity. The following factors are the causes of deviation in this experiment: the relative position of the droplet starting to fall and the substrate, deviations in the volume of the ejected droplet, recognition limitations of droplet motion due to limited frames per second, and the resolution of videos.

#### 3.2.1 Drop Friction Force Instrument (DoFFI)

The friction force acting on the drop was quantified using a hollow rectangular glass capillary sensor. A droplet of fixed volume (20 µL) was attached to the capillary and subsequently moved across the substrate. This approach facilitates precise measurement of the resistance encountered by the droplet as it interacts with the surface. The glass capillary employed for force measurement had dimensions of 0.05 × 0.5 × 50 mm^3^ (CM Scientific Ltd.) and a calibrated spring constant of K = 101.5 µN/mm, with an uncertainty of less than 5%. The spring constant was determined by observing the capillary's free oscillations after applying a small initial displacement. These oscillations were recorded using a CMOS camera. The stage velocity was regulated using an automated X‐Y stage (Krüss DSA100) to ensure uniform and repeatable measurements. Measurements took place at 22°C ± 2°C and 40% ± 10% relative humidity. A side‐view camera was utilized to monitor capillary deflection in real‐time and to conduct a detailed analysis of the advancing and receding contact angles from the same video data. These angles were extracted by processing individual frames using a Python‐based image analysis script. We implemented an adapted version of the 4S‐SROF method to compute measurements like contact angles using the tangent fitting technique [49]. It employs morphological transformations for noise reduction, which have been more effective than traditional median filtering in preserving the droplet's natural shape while also performing well in temporal profiling [50]. For this fitting, 10 pixels on each side were considered, with an image resolution of ≈125 pixels/mm.

## Author Contributions

Junwoo Park conceived the project and jointly supervised the project with Hans‐Jürgen Butt. Dongho Shin and Rutvik Lathia designed and performed the droplet acceleration experiments and surface preparation. Rutvik Lathia conducted friction force measurements using the DoFFI technique. Chirag Hinduja helped in analyzing the data from the DoFFI technique. Hyunbae Cheon contributed to data interpretation and developed the software for analyzing droplet velocity. Junwoo Park, Hans‐Jürgen Butt, Dongho Shin, and Rutvik Lathia wrote the manuscript with contributions from all authors. All authors discussed the results.

## Conflicts of Interest

The authors declare no conflicts of interest.

## Supporting information




**Supporting file**: advs73896‐sup‐0001‐SuppMat.docx

## Data Availability

The data that support the findings of this study are available from the corresponding author upon reasonable request.
