# Supplementary material for: Salt Water Drops Slide Faster: Ionic Modulation of Drop Friction
Source: Adv Sci (Weinh). 2026 Jan 21;13(17):e21659. doi: 10.1002/advs.202521659 (PMC13042842; doi:10.1002/advs.202521659)
Supplement: Supplementary file 1 — Supporting file: advs73896‐sup‐0001‐SuppMat.docx [file ADVS-13-e21659-s001.docx]

**Supplementary Information for**

**Salt Water Drops Slide Faster: Ionic Modulation of Drop Friction**

Dongho Shin,^1,⊥^ Rutvik Lathia,^2,⊥^ Chirag Hinduja,^2^ Hyunbae Cheon,^1^ Seongmin Park,^1^ Hans-Jürgen Butt,^2,*^ and Junwoo Park^1,3,*^

^1^ Department of Chemistry, Sogang University, Mapo-gu, Seoul 04107, Republic of Korea

^2^ Max Planck Institute for Polymer Research (MPI-P), Ackermannweg 10, 55128 Mainz, Germany

^3^ Center for Nano Materials, Sogang University, Seoul 04107, Republic of Korea

⊥ D.S. and R.L. contributed equally to this work.

(*) Author to whom correspondence should be addressed: [parkjw@sogang.ac.kr](mailto:parkjw@sogang.ac.kr) (J.P.) and butt@mpip-mainz.mpg.de (H.-J.B.)

**Figure S1. Experimental setup.** The figure shows the experimental setup. A syringe pump dispenses water droplets onto the surface at regular intervals. A camera records their motion during descent to measure velocity.

**Figure S2. Visualization of the salt-induced acceleration effect.** Sequential snapshots visualizing the movement of a 37 μL DI water droplet (top) and a 37 μL 10^-1^ M NaCl droplet (bottom) on a PFOTS-coated Si substrate (n-type, ρ ≤ 0.005 Ω·cm) at a 50° tilt angle.

**Figure S3. Velocity variation as a function of different electrolytes.** To investigate the effect of different electrolytes, experiments took place on a PFOTS-coated Si surface with a resistivity of ρ ≤ 0.005 Ω·cm. Frictional changes induced by ions extend beyond NaCl and appear as a common characteristic across various electrolytes. All tested water-soluble ions (LiCl, KCl, RbCl, NaF, NaBr) showed similar trends. These findings indicate that the phenomenon arises from a universal effect rather than specific interactions between ions and the solid surface.

**Figure S4. Experimental results on PFOTS-coated Si surfaces with different wafer types.** (a) Velocity as a function of electrolyte concentration on a PFOTS-coated p-type Si surface with a resistivity of ρ ≤ 0.005 Ω·cm. (b) Acceleration variation with concentration on PFOTS-coated n-type and p-type Si surfaces, both with a resistivity of ρ ≤ 0.005 Ω·cm. The results indicate that surface charge carriers have no significant influence, as the phenomenon occurs regardless of the semiconductor carrier type.

**Figure S5. Droplet velocity at varying electrolyte concentrations on PFOTS-coated Si surfaces with different resistivities:** (a) ≤ 0.005 Ω·cm, (b) 1–10 Ω·cm, and (c) > 1000 Ω·cm. A clear increase in velocity with increasing concentration was observed only on highly conductive (low-resistivity) surfaces.

**Figure S6. Surface roughness of the substrates by AFM measurement.** 3D topography images of PFOTS monolayers on (a) Si (ρ ≤ 0.005 Ω·cm) and (b) Si (ρ > 1000 Ω·cm) obtained for an area of 0.1 × 0.1 µm^2^

**Figure S7. Effect of electrolyte concentration on droplet motion across various surfaces:** (a) Lotus leaf, (b) resistive silicon surface, (c) PVDF surface, (d) Teflon surface, and (e) polystyrene (PS) surface.

**Figure S8. Velocity stability of consecutive droplets on PFOTS-coated Si surface.** Experiments tracked the velocity of 100 consecutive droplets on a PFOTS-coated Si surface with a resistivity of ρ ≤ 0.005 Ω·cm using NaCl electrolytes at varying concentrations: (a) DI, (b) 10⁻⁶ M, (c) 10⁻⁵ M, (d) 10⁻⁴ M, (e) 10⁻³ M, (f) 10⁻² M, (g) 10⁻¹ M, and (h) 1 M. Transitioning from DI water to 1 M NaCl caused no significant acceleration differences between the 1^st^, 50^th^, and 100^th^ droplets. The absence of hysteresis indicates that residual charges from contact electrification do not influence the velocity of subsequent droplets.

**Figure S9. Experimental setup for electrical grounding.** As the droplet slides down the surface, it remains electrically connected through a grounded wire, while both the surface and the needle are grounded.

**Figure S10.** **Effect of electrical grounding on droplet motion.** Applying electrical grounding to the droplet, syringe needle, and Si wafer did not stabilize dynamic friction. Instead, higher electrolyte concentrations led to an increase in velocity. This finding confirms that electrical grounding does not influence the acceleration of sliding droplets.

**Figure S11. Velocity variation on Au surfaces with different carbon chain lengths.** Velocity measurements explored the effect of carbon chain length on droplet motion using Au surfaces functionalized with self-assembled monolayers (SAMs) of (a) hexanethiol (C₆H₁₃SH), (b) decanethiol (C₁₀H₂₁SH), (c) tetradecanethiol (C₁₄H₂₉SH), and (d) octadecanethiol (C₁₈H₃₇SH). The results show that velocity enhancement in high-concentration electrolytes decreases as the carbon chain length increases.

**Figure S12. Static contact angle variation on Au surfaces with different carbon chain lengths.** Static contact angle measurements explored the effect of carbon chain length on surface wettability using Au surfaces functionalized with self-assembled monolayers (SAMs) of hexanethiol (C₆H₁₃SH), decanethiol (C₁₀H₂₁SH), tetradecanethiol (C₁₄H₂₉SH), and octadecanethiol (C₁₈H₃₇SH). The results show that the static contact angle increases as the carbon chain length increases, indicating higher hydrophobicity for longer alkyl chains.

**Figure S13. Force and contact angle measurements using scanning drop friction force microscopy (DoFFI).** (a) Measuring surface force in DI water and 1 M NaCl. (b) Evaluating receding contact angle (RCA) and advancing contact angle (ACA) in DI water and 1 M NaCl. These experiments were performed on trichloro(1H,1H,2H,2H–perfluorooctyl)silane (PFOTS)-coated conductive silicon wafer surfaces. The results show that dynamic friction between the droplet and the surface decreases in 1 M NaCl compared to DI water. In addition, RCA increases and ACA decreases in 1 M NaCl, indicating lower contact angle hysteresis (CAH) and reduced interfacial friction. This trend matches the reduction in friction.

**Figure S14. Velocity enhancement induced by KNO₃ electrolyte.** (a) Velocity variation as a function of electrolyte concentration was examined on a PFOTS-coated Si surface with a resistivity of ρ ≤ 0.005 Ω·cm. (b) Acceleration trends were analyzed across different concentrations. Droplet velocity increased with KNO₃ electrolyte, demonstrating a clear dependence on electrolyte concentration.
